# Airway Management Challenges During General Anesthesia in a Patient With a Large Lingual Cavernous Hemangioma: A Case Report

**DOI:** 10.7759/cureus.104039

**Published:** 2026-02-21

**Authors:** Apostolos Ntanasis, Elisavet Melissi, Aikaterini Ntaflou, Evangelos Sitos, Dimitrios Alefragkis, George Mpourazanis, Petros Papalexis, Nikolas Matthaiou, Christos Akrivis, Freideriki Steliou

**Affiliations:** 1 Department of Anesthesiology, General Hospital of Ioannina G. Hatzikosta, Ioannina, GRC; 2 4th University Department of Internal Medicine, Hematology-Oncology Unit, University General Hospital Attikon, Haidari, GRC; 3 Department of Nursing, School of Health Sciences, National and Kapodistrian University of Athens, Athens, GRC; 4 Department of Obstetrics and Gynecology, General Hospital of Ioannina G. Hatzikosta, Ioannina, GRC; 5 First Department of Internal Medicine, Endocrinology Unit, Laiko General Hospital, National and Kapodistrian University of Athens, Athens, GRC; 6 Interventional Radiology Unit, AHEPA University General Hospital, Thessaloniki, GRC; 7 Department of Medicine, School of Medicine, Aristotle University of Thessaloniki, Thessaloniki, GRC

**Keywords:** difficult airway management, general anesthesia, hemangioma, lingual cavernous hemangioma, video laryngoscopy (vl)

## Abstract

The induction of general anesthesia in patients with airway compromise poses significant challenges, particularly in the presence of oral vascular tumors like hemangiomas, which are benign but can lead to substantial bleeding. This case study discusses an 82-year-old woman with endometrial carcinoma scheduled for elective total hysterectomy with bilateral salpingo-oophorectomy. Preoperative evaluation revealed a large lingual cavernous hemangioma. Key anesthetic considerations included difficulties with mask ventilation and intubation, bleeding from the hemangioma, and the risk of blood aspiration. However, the induction of anesthesia, along with mask ventilation and intubation facilitated by a video laryngoscope, was executed without complications, leading to successful extubation. On the fifth postoperative day, the patient was discharged following an uncomplicated recovery. A comprehensive airway management strategy for patients with oral cavity hemangioma enables successful intubation and general anesthesia while minimizing the necessity for invasive or rescue airway methods, underscoring the importance of strategic airway management in these scenarios.

## Introduction

The difficult airway due to comorbidities or anatomical variations is one of the challenges that trained anesthesiologists are required to deal with in their everyday clinical practice. Challenges spiral in the case of lingual hemangiomas, whose large size and uncontrolled bleeding limit airway patency during mask ventilation, intubation, and/or pulmonary aspiration for general anesthesia [1,2]. Intramuscular hemangiomas are benign vascular tumors most commonly affecting the head and neck region. The oral cavity, including the lips, buccal mucosa, and tongue, is frequently involved. Invasive muscular hemangiomas are even rarer (<1% of the total), and massive cavernous variations are sporadically described. Literature reports limited cases of large cavernous lingual hemangiomas and even fewer regarding airway management [3-6]. Hemangiomas are vascular malformations commonly encountered in infants and adolescents, with cavernous hemangiomas affecting approximately 1 in every 200 individuals. Only 10% of affected individuals exhibit symptoms despite disease prevalence. Histological classification of hemangiomas includes the capillary and cavernous subtypes, with mixed tumors being more common. Although predominantly congenital, hemangiomas can also develop in adulthood. Head and neck lesions are relatively rare, particularly in the mouth and radix lingue regions [3,7]. Diagnosis prerequisites differentiate them from other vascular and tumoral lesions; these often manifest during early childhood but can be misdiagnosed as multiple benign or malignant entities, including enteric duplication cyst, thyroglossal duct cyst, vallecular cyst, lymphatic malformation, sarcoma, schwannoma, Burkitt lymphoma, and other conditions [7]. We report a rare case of an incidental cavernous lingual hemangioma in a female patient detected following neck magnetic resonance imaging (MRI). We discuss its management by the anesthesiological team for intubation-induced general anesthesia prior to elective resection.

## Case presentation

An 82-year-old woman was referred to the obstetrics and gynecology department for elective total abdominal hysterectomy with bilateral salpingo-oophorectomy (THBSO) due to FIGO grade II endometrial carcinoma [8]. Her preoperative vital signs were stable, with a recorded blood pressure of 125/80 mmHg, a temperature of 36.6°C, a pulse rate of 87 beats per minute, a respiratory rate of 17 breaths per minute, and an oxygen saturation level of 95%. Notably, the patient had never undergone a Pap smear test, and her obstetric history included two vaginal deliveries, with no reported abortions or miscarriages. This clinical presentation underscores the evaluation and management of elderly patients with gynecologic cancers, highlighting the importance of thorough preoperative assessments and consideration of individual medical histories.

Her medical regimen includes irbesartan 150 mg, aspirin 100 mg, a combination of sitagliptin 50 mg and metformin 1000 mg, dapagliflozin 10 mg, and a combination of fenofibrate-simvastatin 145/20 mg along with amlodipine 10 mg, all administered once daily. These are for her long-standing type II diabetes mellitus, hypertension, and hyperlipidemia. She reported no allergy history. There were no smoking habits or alcohol use. Her body mass index (BMI) was measured at 32 kg/m², obese class I. Her surgical history notes a left-sided partial thyroidectomy at age 77 for a cold-on-scintigraphy adenomatous nodule, for which she is treated with levothyroxine 0.1 mg and monitored biannually through clinical evaluation and neck ultrasound. No pertinent family history was reported.

Preoperative clinical evaluation revealed no significant abnormalities apart from age-consistent grade I left ventricular diastolic dysfunction [9]. All laboratory tests returned normal results after the THBSO operation (Table 1). 

**Table 1:** Pre-operative and post-operative laboratory results for total abdominal hysterectomy with bilateral salpingo-oophorectomy (THBSO) N/A: Not available; aPTT: activated partial thromboplastin time; HBG: hemoglobin; HCT: hematocrit; PLT: platelet

Parameter	Day 0 (Admission Day)	Day 1 (Operation Day)	Day 3	Day 5 (Exit Day)	Follow-up (60 Days)	Reference Values
WBC	11.68 k/μL	13.14 k/μL	13.74 k/μL	11.2 k/μL	9.5 k/μL	4-11 k/μL
Neutrophils	52,00%	58.7 %	66.3 %	68.1%	58.9%	40-75 %
LYMPH	39.2%	32.3 %	25.4 %	26.4 %	22.2%	20-45 %
HBG	13.3 g/dL	11.2 g/dL	12.1 g/dL	12.9 g/dL	14 g/dL	11.8-17.8 g/dL
HCT	40.4 %	34.3 %	36.8%	37.8 %	41.5 %	36-52 %
INR	1.06	1.05	N/A	N/A	N/A	0.8-1.2
aPTT	32.90 sec	31.21 sec	N/A	N/A	N/A	26-36 sec
PLT	400 k/μL	342 k/μL	298 k/μL	394 k/μL	410 k/μL	140-450 k/μL
GLC	133 mg/dL	94 mg/dL	110 mg/dL	99 mg/dL	85 mg/dL	70-115 mg/dL
URE	33 mg/dL	29 mg/dL	20 mg/dL	30 mg/dL	25 mg/dL	10-50 mg/dL
CRE	0.86 mg/dL	0.78 mg/dL	0.81 mg/dL	0.90 mg/dL	0.6 mg/dL	0.5-1.1 mg/dL
K+	4.7 mmol/L	4.1 mmol/L	4.4 mmol/L	4.2 mmol/L	3.9 mmol/L	3.5-5.1 mmol/L
Na+	140 mmol/L	138 mmol/L	140 mmol/L	142 mmol/L	140 mmol/L	136-146 mmol/L
TPR	6.4 g/dL	5.7 g/dL	6.7 g/dL	7 g/dL	6.2 g/dL	6.2-8.4 g/dL
ALB	4.5 g/dL	3.5 g/dL	4.0 g/dL	4.4 g/dL	3.8 g/dL	3.5-5.1 g/dL
AST	39 U/L	26 U/L	30 U/L	29.2 U/L	17 U/L	5-33 U/L
ALT	14 U/L	17 U/L	25 U/L	23 U/L	17 U/L	5-32 U/L
GGT	5 IU/L	5 IU/L	15 IU/L	24 IU/L	10 IUL	5-31 IU/L
UA	4.1 mg/dL	4.2 mg/dL	2.8 mg/dL	3 mg/dL	2.5 mg/dL	2.3-6.1 mg/dL
TSH	1.23	N/A	N/A	N/A	1.30	0.35-4.94 μIU/mL
FT4	1.00	N/A	N/A	N/A	1.15	0.70-1.48 ng/dL
FT3	2.02	N/A	N/A	N/A	2.15	1.88-3.18 pg/mL

**Figure 1:**
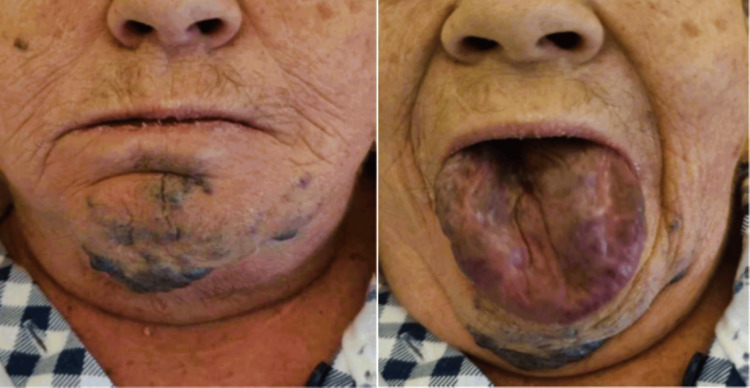
Frontal face view of the patient exhibiting tongue protrusion

A notably large lingual lesion, observed during the preoperative anesthesiological assessment and clinical examination, was reported to have been present since birth without any size changes. The ASA physical classification was determined before anesthesia, taking into account her age, obesity reflected in her BMI, and her medical history, resulting in an ASA III classification. Visualization of the hypopharynx was inadequate during the assessment of oropharyngeal and vocal cord visibility, categorizing it as Mallampati class IV [10]. Macroscopic examination of the lesion revealed it to be exophytic, measuring 5 cm at its largest diameter, protruding from the tongue, and extending into the hypopharynx. The lesion elevated the mucosal surface but showed no indications of invading nearby structures (Figure 1). 

**Figure 2:**
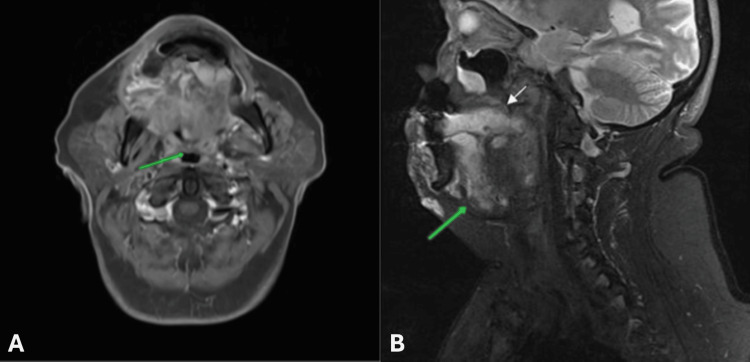
Head and neck magnetic resonance imaging (MRI) findings of a large lobulated mass extending across the entirety lingual dorsum (presulcal and postsulcal) with substantial inhomogeneous enhancement following intravenous (IV) contrast administration (A): The mass was hypointense on axial T1 fluid-attenuated inversion recovery (FLAIR) sequences, and oropharyngeal airway narrowing was displayed (green arrow). (B): Sagittal T2 sequences revealed a hyperintense lesion with multiple small hypointense foci compatible with flow voids, abutting the palatinum cranially (white arrow) and the left of the lingual dorsum caudally (green arrow).

Previous imaging was not available for comparison, and a head and neck MRI scan was ordered to assess mass origin and relationship with surrounding tissues (Figure 2). 

In this case, the anesthesia management began with aspiration prophylaxis, which consisted of the intravenous (IV) administration of ondansetron at 4 mg to prevent nausea and omeprazole at 40 mg to inhibit gastric acid secretion. Pre-oxygenation was conducted for a duration of three minutes using a large anatomical face mask delivering 100% oxygen. Following this, premedication included the administration of 50 μg of IV fentanyl for analgesia. For the induction of anesthesia, propofol was administered either at a dosage of 3 mg/kg or a total of 200 mg, complemented by the administration of rocuronium at a dose of 1 mg/kg or 70 mg to facilitate muscle relaxation. Satisfactory mask ventilation was ensured throughout the procedure. Intubation was performed using a video laryngoscope, which allowed for minimal manipulation to reduce the risk of massive bleeding. For airway management, an 8.0 mm internal diameter endotracheal tube was utilized. During the maintenance phase of anesthesia, sevoflurane was employed as the inhalation agent at a concentration of 1-2% MAC (minimum alveolar concentration) between 0.8 and 1.2. A continuous fentanyl infusion was maintained until the abdominal cavity was opened, amounting to a total of 250 μg of fentanyl. Additionally, fresh gas flow was maintained with a mixture of oxygen and air in a 50:50 ratio, ensuring adequate ventilation throughout the surgical procedure.

The patient underwent a successful extubation without complications, exhibiting adequate spontaneous respiration and responsiveness, including eye-opening to verbal commands. Following the completion of a THBSO procedure, the patient proceeded to postoperative recovery and was subsequently transferred to the post-anesthesia care unit as intended. The discharge occurred on the fourth day after the surgery, which is documented in the patient's morbidity record.

## Discussion

Airway management

The management of airway during intubation procedures is critical and revolves around three key components: the identification of potential peri-intubation complications, a thorough assessment of foreseeable risks, and the availability of hospital equipment. In this instance, the patient was positioned supinely in a manner that minimized the risk of inducing dyspnea, successfully maintaining an oxygen saturation level of 96% while breathing ambient air. Standard monitoring protocols were established, which included continuous five-lead electrocardiography (ECG), heart rate monitoring, and non-invasive blood pressure measurements to ensure the patient's stability throughout the procedure. Important anesthetic considerations were highlighted, specifically the risks associated with difficult mask ventilation and potential intubation challenges. Additionally, concerns regarding the bleeding risk from any existing hemangiomas were acknowledged, alongside the possibility of blood aspiration. To facilitate a comprehensive airway assessment during the pre-anesthetic check-up, the LEMON score framework, comprising elements of Look, Evaluate, Mallampati grade, Obstruction, and Neck mobility, was employed to guide clinical decision-making effectively [11].

Oncological management

Treatment is generally dispensable for lingual hemangiomas presenting at a young age, as they tend to regress spontaneously. However, 10-20% may require intervention depending on patient age, clinical manifestations, and individual anatomical considerations. Common treatment strategies include surgical resection, embolization, corticosteroid therapy, sclerotherapy, and laser photocoagulation. Aggressive treatment is reserved for cases with severe complications such as airway obstruction, dysphagia, and bleeding. Surgical treatment ranges from simple excision for small lesions to extensive resection for larger and/or deeper-seated tumors. Recurrence is possible and necessitates regular follow-up, despite no consensus on the optimal frequency to date [12-16].

Airway management challenges and hemorrhage risk

Airway management in patients with intraoral hemangiomas poses significant challenges that include difficult ventilation and intubation and an increased risk of bleeding and aspiration. Patients with large lingual cavernous hemangiomas often exhibit significant anatomical distortion that complicates airway management [1,2]. In our case, preoperative evaluation revealed Mallampati class IV and Cormack-Lehane class II, indicating a high risk of difficult intubation. These findings necessitated careful planning and advanced airway management techniques. Fiber-optic nasotracheal intubation is preferred but requires expertise and availability. Video laryngoscopy with the video laryngoscope was deemed effective and revealed Cormack-Lehane class II [17], enabling airway visualization with minimal manipulation and reducing the risk of mass-derived hemorrhage. This approach conforms to contemporary difficult airway management recommendations, emphasizing the importance of structure visualization and the use of video-assisted devices. Prompt extubation was feasible by virtue of short surgery duration and minimal anticipated edema [18,19]. The pronounced vascularity of hemangiomas poses a substantial risk of hemorrhage that may be further exacerbated by impetuous airway manipulation. Employing video-assisted laryngoscopy techniques minimizes direct contact with the lesion and mitigates the risk of trauma and subsequent bleeding. Blood product availability and an experienced, provident, and prescient surgical team are also crucial for managing potential intraoperative hemorrhage [1].

Perioperative anesthetic considerations 

Although neuraxial anesthesia may be considered for abdominal hysterectomy in selected patients, general anesthesia was intentionally chosen in this case. The presence of a large intraoral vascular lesion posed a potential risk of bleeding or sudden airway compromise. Securing the airway in a controlled and elective setting prior to surgical stimulation was considered safer than risking emergent airway intervention under neuraxial anesthesia. Furthermore, the anticipated duration and extent of the abdominal procedure favored general anesthesia for optimal surgical conditions, hemodynamic control, and patient comfort. Therefore, planned general anesthesia with structured airway preparation was deemed the most appropriate and safest strategy. Preoperative preparation is critical for managing patients with large lingual cavernous hemangiomas. Administering aspiration prophylaxis, ondansetron, and omeprazole, and carefully selecting appropriate induction agents (propofol and rocuronium) ensures smooth induction while maintaining stable hemodynamics and adequate muscle relaxation. Pre-oxygenation with 100% oxygen is essential to mitigate the risk of hypoxia during intubation [1]. Continuous vital sign monitoring (such as oxygen saturation, heart rate, and blood pressure) is indispensable. Maintenance with sevoflurane was supplemented by fentanyl infusion and balanced fresh gas flow, which provided stable anesthesia and ensured patient safety in our case. Anesthetic agent selection should be adjusted for hemodynamic effects, tailored to individual patient characteristics, and based on the capacity to assist prompt recovery and facilitate postoperative neurological function evaluation [20]. Adequate spontaneous respiration, consciousness, and airway protection must be ensured for patients operated on for large lingual hemangiomas to be extubated. We extubated once the above conditions were met and complications such as airway obstruction or aspiration were avoided. It is recommended to monitor in a high-dependency unit postoperatively until the patient is fully awake and the risk of airway compromise has subsided [21]. All airway assessment tools mentioned, including the Mallampati classification, LEMON score, and Cormack-Lehane classification, are freely available clinical tools that do not require licensing and are reported in Table 2. 

**Table 2:** Tools and classification systems (Mallampati Classification, LEMON Score, Cormack-Lehane Classification and video laryngoscopy) All tools are freely available, non-copyrighted, and do not require a license.

Tool/Classification/References	Purpose	Patient's Findings	License/Permission
Mallampati Classification (Class I-IV) [10]	Preoperative assessment of the oropharyngeal anatomy is essential for predicting difficult intubation. The visibility of anatomical structures is classified into four classes: Class I indicates full visibility of the soft palate, uvula, fauces, and pillars. Class II shows visibility of the soft palate and the majority of the uvula and fauces. Class III demonstrates visibility of the soft palate and the base of the uvula. Class IV is characterized by visibility of only the hard palate.	Mallampati Class IV	No license is required for the freely available clinical classification
LEMON Score (Look Externally, Evaluate, Mallampati, Obstruction, Neck mobility) [11]	Structured pre-anesthetic assessment of a difficult airway involves several evaluations: L for Look externally, inspecting for facial trauma or anatomical features. E for Evaluate using the 3-3-2 rule to measure mouth opening and distances related to the hyoid and thyroid. M for Mallampati score, where Classes 3 or 4 indicate difficult intubation. O for Obstruction, identifying conditions that may obstruct the airway, and N for Neck mobility, assessing the ability to extend the neck.	A high-risk airway is indicated by visible obstruction and limited oropharyngeal visualization	No license is required for the freely available clinical classification
Cormack-Lehane Classification (Grade I-IV) [17]	Intraoperative grading during laryngoscopy consists of four grades: Grade I indicates a full view of the glottis (vocal cords). Grade II signifies a partial view with subdivisions IIa (partial view) and IIb (only arytenoids visible). Grade III reveals only the epiglottis while the glottis remains hidden, and Grade IV reflects no visibility of either the epiglottis or glottis.	Grade II view using video laryngoscopy	No license is required for the freely available clinical classification
Video laryngoscopy [19]	Airway visualization and facilitation of tracheal intubation	Successful intubation with minimal manipulation and no bleeding	Medical device: Not a system of points. No license is needed to report

## Conclusions

This case highlights the critical need for anesthesiologists to maintain heightened awareness and preparedness when dealing with patients who have rare upper airway vascular lesions. With advancements in imaging and video-assisted airway techniques, anesthesiologists can more effectively anticipate and manage complex airway situations that previously posed high risks. Future practices should focus on structured airway assessments, early collaboration among multidisciplinary teams, and the use of new airway technologies to reduce morbidity related to airway interventions. Sharing and reporting similar cases will help build a robust evidence base to enhance training and guidelines, ultimately improving patient safety and outcomes. For patients with suspected or confirmed oral cavity hemangiomas, customized anesthetic strategies and careful airway management are essential for successful perioperative care.
